# Clinical Pathobiochemistry of Vitamin B_12_ Deficiency: Improving Our Understanding by Exploring Novel Mechanisms with a Focus on Diabetic Neuropathy

**DOI:** 10.3390/nu15112597

**Published:** 2023-06-01

**Authors:** Erwin Schleicher, Triantafyllos Didangelos, Evangelia Kotzakioulafi, Alexander Cegan, Andreas Peter, Konstantinos Kantartzis

**Affiliations:** 1 Institute for Clinical Chemistry and Pathobiochemistry, Department for Diagnostic Laboratory Medicine, University Hospital of Tübingen, 72076 Tübingen, Germany; erwin.schleicher@med.uni-tuebingen.de (E.S.); andreas.peter@med.uni-tuebingen.de (A.P.); 2 Institute for Diabetes Research and Metabolic Diseases of the Helmholtz Center Munich, German Center for Diabetes Research (DZD), 72076 Tübingen, Germany; 3 German Center for Diabetes Research (DZD e.V.), 85764 Neuherberg, Germany; 4 Diabetes Center, 1st Propaedeutic Department of Internal Medicine, Medical School, “AHEPA” Hospital, Aristotle University of Thessaloniki, 54621 Thessaloniki, Greece; didang@auth.gr (T.D.); ekotzaki@auth.gr (E.K.); 5 Department of Biological and Biochemical Sciences, Faculty of Chemical Technology, University of Pardubice, 53210 Pardubice, Czech Republic; alexander.cegan@upce.cz; 6 Department of Internal Medicine IV, Division of Endocrinology, Diabetology and Nephrology, University of Tübingen, 72076 Tübingen, Germany

**Keywords:** cobalamin, diabetes, homocysteine, methylmalonic acid, peripheral neuropathy, laboratory biomarker, oxidative stress, reactive oxygen species, redox, Vitamin B_12_

## Abstract

Vitamin B_12_ (B_12_) is an essential cofactor of two important biochemical pathways, the degradation of methylmalonic acid and the synthesis of methionine from homocysteine. Methionine is an important donor of methyl groups for numerous biochemical reactions, including DNA synthesis and gene regulation. Besides hematological abnormalities (megaloblastic anemia or even pancytopenia), a deficiency in B_12_ may cause neurological symptoms, including symptoms resembling diabetic neuropathy. Although extensively studied, the underlining molecular mechanism for the development of diabetic peripheral neuropathy (DPN) is still unclear. Most studies have found a contribution of oxidative stress in the development of DPN. Detailed immunohistochemical investigations in sural nerve biopsies obtained from diabetic patients with DPN point to an activation of inflammatory pathways induced via elevated advanced glycation end products (AGE), ultimately resulting in increased oxidative stress. Similar results have been found in patients with B_12_ deficiency, indicating that the observed neural changes in patients with DPN might be caused by cellular B_12_ deficiency. Since novel results show that B_12_ exerts intrinsic antioxidative activity in vitro and in vivo, B_12_ may act as an intracellular, particularly as an intramitochondrial, antioxidant, independent from its classical, well-known cofactor function. These novel findings may provide a rationale for the use of B_12_ for the treatment of DPN, even in subclinical early states.

## 1. Introduction

Vitamin B_12_ (B_12_) is an essential cofactor for two enzymes in human metabolism: methylmalonyl-CoA mutase (catalyzing the conversion of methylmalonyl-CoA to succinyl-CoA), and methionine synthase (catalyzing the synthesis of methionine from homocysteine). While an inherited defect of methylmalonyl-CoA mutase causes methylmalonic aciduria, severe acquired B_12_ deficiency, mostly due to reduced uptake of B_12_, causes classical pernicious anemia. It may also cause neurological symptoms, most commonly sensory, but also motoric or painful neuropathy, symptoms that are also common in DPN.

The precise molecular mechanism leading to DPN is under debate [1,2,3,4]. Previous reviews implicate a variety of hyperglycemia-dependent pathways, including the sorbitol, glucosamine and the AGE/RAGE pathways, possibly leading to oxidative stress and induction of inflammatory pathways [5]. However, none of these pathways have been unequivocally shown to be causally responsible for the development of DPN. To date, other than glycemic control, there is no evidence-based therapy available which may prevent DPN [5,6,7,8]. Numerous reports indicate beneficial effects of B_12_ supplementation, while other studies did not find significant effects [9,10]. In a recent extensive review, the therapeutic effects of B_12_ on DPN were evaluated, and significant effects of B_12_ on DPN could be demonstrated [10]. However, no molecular mechanism has been hitherto established for the beneficial effects of B_12_ in the prevention or treatment of DPN. Furthermore, it is not known which B_12_ levels should be considered as indicating B_12_ deficiency (and thus needing B_12_ supplementation). Indeed, B_12_ serum concentrations observed in different states of DPN may be only marginally reduced or even unchanged. In addition, elevated homocysteine and methylmalonic acid levels, both biomarkers of cellular B_12_ deficiency, are common in the elderly and are associated with neurological abnormalities [11]. It was suggested that a functional B_12_ deficiency is present in these patients, despite normal B_12_ serum levels. This would mean that intracellular B_12_ does not always reflect serum levels. Notably, patients with DPN do not always show signs of classical B_12_ deficiency, e.g., pernicious anemia, which suggests that DPN may develop at lower cellular (and not necessarily lower serum) levels of B_12_. It should be underlined that subclinical B_12_ deficiency may develop very slowly because the liver contains large amounts of B_12_; thus, clinical signs develop slowly and may be overlooked.

Accordingly, in this review, we first summarize the current knowledge of the possible involvement of B_12_ deficiency in the development of DPN and provide novel data introducing the property of B_12_ working as redox system. Second, we discuss the issue and the implications of “functional” or “subclinical” B_12_ deficiencies. For the detection of such subclinical B_12_ deficiencies, cut-off values need to be defined. Such cut-off values should include cut-off values for overt B_12_ deficiency, ranges for inadequate B_12_ supply and values for adequate supply.

## 2. Biochemistry and (Patho)-Physiology

- Chemistry;
- Vitamin B_12_ sources, physiological uptake and causes of deficiency;
- Intracellular processing and reduction/oxidation function of B_12_;
- Physiological functions of Vitamin B_12_;
- Clinical pathophysiology of B_12_ deficiency.

### 2.1. Chemistry

**Figure 1:**
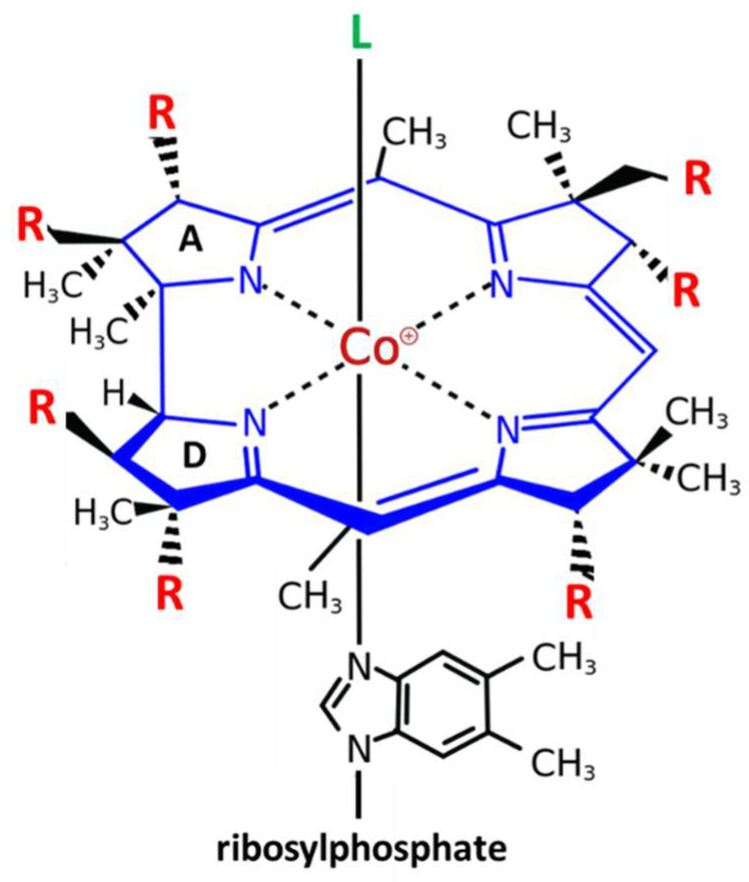
Structure and modifications of Vitamin B_12_ (cobalamin). Cobalamin (Cbl) is the only cobalt containing vitamin. The central cobalt atom (magenta) is coordinated by a corrin ring (blue) with 7 residues (R = acetyl or propionylamide) (red). Only the upper axial position (L) (green) can be exchanged for different ligands: the physiological ligands 5′-desoxyadenosyl-Cbl or methyl-Cbl, which are needed for two different biochemical reactions, or the pharmacologically administered forms of B_12_ cyano-Cbl (more in USA) or hydroxy-Cbl (more in Europe). The unique characteristic of B_12_ is that a single cobalt atom is bound in the center of a ring of four pyrroles (corrin) similar to the Fe atom bound in the center of the heme ring of hemoglobin The only difference between the tetrapyrrolic ring of corrins and heme is that the A and D pyrroles are directly bound in corrins, while in heme, the four rings are all bound via a carbon bridge.

Vitamin B_12_ or cobalamin is a water-soluble vitamin with a complex structure (Figure 1). The unique characteristic of B_12_ is that a single cobalt atom is bound in the center of a ring of four pyrroles (corrin) similar to the Fe atom bound in the center of the heme ring of hemoglobin. Accordingly, similar to the heme system of hemoglobin where the upper axial ligand O_2_ can be easily exchanged, the upper axial ligand of B_12_ is also exchangeable. Physiological ligands of B_12_ are a methyl (-CH_3_), hydroxy (-OH) or a 5’-desoxyadenosyl unit [12]. Between them, hydroxy-B_12_ is the more stable form, and is used, as well as cyano-B_12_, for pharmacological administration of B_12_ in Europe and the US, respectively (Figure 1). 

### 2.2. Vitamin B_12_ Sources, Physiological Uptake and Causes of Deficiency

B_12_ is not present in foods obtained from plants, and since it cannot be produced by humans, it needs to be obtained from a diet containing products derived from animals, such as meat, dairy products and particularly liver, which is the most important B_12_ storage organ (80% of total storage) in mammals. The recommended daily requirement depends on gender. Adult women require 2.4 µg of B_12_ per day. This requirement increases to 2.8 µg of B_12_ per day during pregnancy and breastfeeding. Adult men can meet their needs by consuming 2.6 µg of B_12_ per day. Lesser amounts are recommended for young infants [13]. The daily turnover of B_12_ is less than 0.1%. Deficiencies occur when supplies are reduced to 300 µg of B_12_ per day. As mentioned above, B_12_ deficiency may be easily overlooked: the clinical signs of deficiency may develop slowly because in humans, B_12_ storage ranging from 2 to 5 mg may last for more than a year.

The sequential stages of uptake of B_12_ (Table 1, left column) [3,14] are as follows: (1) The first step is dietary intake of free or protein-bound B_12_. (2) In the stomach gastric cells release proteases, e.g., pepsin and HCl, and in this acidic milieu, protein-bound B_12_ is released by protein digestion and free B_12_ is bound by haptocorrin. This complex and intrinsic factor (IF) secreted by parietal cells are transferred to the duodenum. (3) In the duodenum, haptocorrin-bound B_12_ (also named “transcobalamin I”) is released from haptocorrin by proteolytic digestion, and the released B_12_ binds to IF with high affinity. (4) The B_12_–IF complex is bound and taken up by specific mucosal receptors in the distal ileum. (5) After internalization, B_12_ is released and free B_12_ is secreted into the blood, bound to transcobalamin and transported to peripheral organs/targets. (6) B_12_ bound to transcobalamin II (HoloTC) is taken up via the ubiquitous receptor CD 320, and B_12_ is liberated in the lysosomes. Note that only about 10–20% of B_12_ is bound to transcobalamin II (HoloTC) in the blood, while the majority of B_12_ (ca. 80%) is bound to transcobalamin I. The latter complex cannot be taken up by peripheral cells. Therefore, determination of HoloTC is superior to the traditional determination of total B_12_, i.e., B_12_ bound to both transcobalamin II and transcobalamin I (see Section 4.1).

Possible defects that may lead to B_12_ deficiency are listed in the right column of Table 1 [1,2,3]. (1) Inadequate B_12_ supply, either due to a strict vegan diet or eating disorders, may cause B_12_ deficiency. (2) Reduced acidification in the stomach, due to aging or drugs (Proton Pump Inhibitors or H_2_-Receptor antagonists) may lead to reduced release of B_12_. The term “Food cobalamin malabsorption” is used to describe these cases of B_12_ deficiency when normal amounts of B_12_ are ingested with food but the vitamin cannot be released from dietary proteins. These individuals can absorb B_12_ from supplements in which the vitamin is not protein-bound or when B_12_ is administered as a drug. Food cobalamin malabsorption is common among older people, in whom the resulting B_12_ deficiency is, in many cases, “subclinical”, i.e., featuring no clinical signs, such as megaloblastic anemia. (3) One of the most common causes is an insufficiency of the parietal cells, which may have been destroyed by autoimmune mechanisms or resected by gastrectomy. This autoimmunity can be diagnosed by detecting autoantibodies against parietal cell and/or IF. For the detection of such atrophic gastritis, anti-IF antibodies are more specific than anti-parietal cell antibodies (100% vs. 90%), but their sensitivity is much lower (37% vs. 81%) [15]. (4) An important cause of reduced resorption of B_12_ are forms of malabsorption which may be caused by surgery or inflammatory bowel diseases (M. Crohn or Colitis ulcerosa) involving the distal ileum. Furthermore, numerous observational and interventional studies, including meta-analyses, indicate that the use of metformin may reduce B_12_ bioavailability [9,16,17,18,19,20,21,22]. The strongest evidence comes from a randomized clinical trial reported by De Jager et al. [23]. Although there are indications that metformin reduces the B_12_ uptake in the terminal ileum [16,20,22], the exact molecular mechanism by which chronic metformin treatment may cause B_12_ deficiency remains unclear [9,16,17,24,25]. Different mechanisms have been suggested: (i) Metformin interferes with the absorption of B_12_ by impairing the calcium-dependent binding of the IF–B_12_ complex to the cubilin receptor on enterocytes. (ii) Metformin might enhance hepatic B_12_ accumulation, thereby altering B_12_ tissue distribution and metabolism. (iii) Metformin may interfere with the reabsorption of bile acids in the enterohepatic circulation because some B_12_ is excreted in bile and may not be reabsorbed under this condition [26]. Considering that nearly all diabetic patients take metformin and many also proton pump inhibitors (mostly for gastric protection because of concomitant use of aspirin or other antiplatelets for cardiovascular complications of diabetes), it is not surprising that B_12_ deficiency is common in diabetic patients. (5) Congenital defects of transport proteins (transcobalamins) or (6) congenital defects of intracellular transport or processing may cause B_12_ deficiency with the respective clinical signs. 

### 2.3. Intracellular Processing and Reduction/Oxidation Function of B_12_

**Figure 2:**
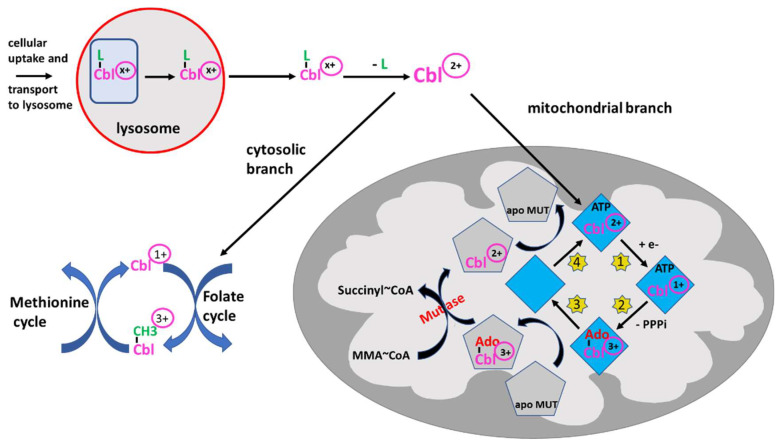
Intracellular processing of B_12_ (Cbl) and redox activity. B_12_ -loaded HoloTC is taken up via receptor-mediated endocytosis in peripheral cells and transported to the lysosome. Since the oxidation state of B_12_/Cbl is dependent on the ligand, -CN, -OH or -CH_3_, the oxidation state is indicated by (x+). After degradation of the transport protein, free Cbl is transported into the cytosol where Cbl^x+^ is “denuded” from the ligand by the β-ligand transferase activity, thus being converted to Cbl^2+^ and entering the branching point: (i) either it remains in the cytosol and enters the methionine cycle for the synthesis of methionine, as outlined in the figure, undergoing reduction and oxidation reactions from 3+ to 1+ and vice versa, or (ii) B_12_ is transported into the mitochondria by a still unknown way to serve as cofactor of methylmalonyl mutase (MUT). After binding to adenosyl transferase (ATR) (blue rhombus), Cbl^2+^ is reduced to Cbl^1+^ (step 1, yellow stars). In a second step (yellow star 2), by using ATP, Cbl is adenosylated (Ado-Co^3+^) and triphosphate (PPPi) is liberated. This complex can transfer adenosylated Cbl to apo MUT (grey pentagon) (step 3). The MUT loaded by adenosylated Cbl^3+^ can catalyze the conversion of methylmalonyl-CoA (MMA-CoA) to succinyl-CoA, which may be metabolized further via the tricarbonic acid cycle. The Cbl off-loaded ATR binds ATP and is regenerated by taking up Cbl^2+^ from inactive MUT (step 4). The reaction schemes indicate the reduction/oxidation versatility of B_12_. Several auxiliary steps are omitted for clarity.

The sophisticated intracellular transport and processing of B_12_ deserves detailed attention. Not only because multiple (but rare) inherited defects have been found causing various forms of B_12_ deficiency, as extensively reviewed by Froese DS et al. [27], but also because these results have elucidated the properties of B_12_ acting as redox system: B_12_ bound to HoloTC is taken up by receptor mediated endocytosis. This complex is then directed to the lysosomes, degraded, and free B_12_ is then transported into the cytosol (Figure 2) [28]. Within the cytosol, B_12_ is processed by several proteins and the central cobalt is reduced to Co^2+^ to facilitate the exchange of the axial ligand, e.g., hydroxy- or methyl-B_12_, for the appropriate ligands needed [27]. The final B_12_-protein complex is then targeted to the cytosolic methionine synthase and the active form, methyl-B_12_, is formed by reduction of the central cobalt of B_12_ to Co^1+^. Methyl-B_12_ presents the active cofactor of methionine synthase. The physiological function of B_12_ in methionine synthase is discussed below.

If bound to other protein(s) containing a mitochondrial leader sequence, B_12_ is targeted to the mitochondria where the methylmalonyl-CoA mutase resides [29]. In the mitochondria, B_12_ is further processed by adenosyl transferase (ATR), an enzyme catalyzing the ATP-dependent synthesis of adenosyl-B_12_(Co^1+^), which is then transferred to methylmalonyl-CoA mutase (MUT), yielding the active enzyme (Figure 2) [30,31]. Together, these data show that B_12_ can be processed and cycled to different oxidation states, i.e., with Co^1+^, Co^2+^ or Co^3+^, in the cytosol and mitochondria as well. A comprehensive overview of the clinical characteristics, treatments and outcomes of nutritional and acquired B_12_ deficiencies, impairments in B_12_ absorption and intracellular trafficking has been previously published [32].

### 2.4. Physiological Functions of Vitamin B_12_

**Figure 3:**
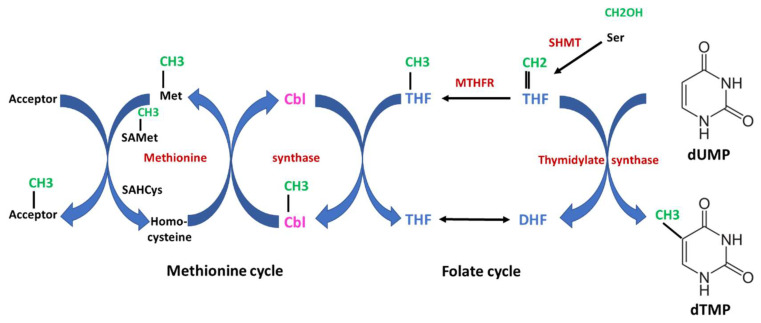
Interrelation of Vitamin B_12_ (cobalamin) and folate metabolism. The scheme shows an overview of the one-carbon metabolism. The two essential vitamins, B_12_ and folate, are important for the transfer of one-carbon units. Both the folate cycle and the methionine cycle are tightly interrelated. In the first step catalyzed by serine hydroxymethyltransferase (SHMT), a one-carbon unit is transferred from serine to tetrahydrofolate (THF), yielding 5,10-methylentetrahydrofolate which is subsequently converted to methyl-THF by methylentetrahydrofolate-reductase (MTHFR). This reaction is irreversible. The one-carbon (methylen- or methyl-) units are highlighted in green, enzymes in red and cobalamin (Cbl) in magenta. Starting from methyl-THF to the left, the methyl unit is transferred to Cbl to form methyl-Cbl, which is further transferred to homocysteine, yielding methionine. Both steps are catalyzed by methionine synthase. Together, the methyl-group is transferred from methyl-THF to methionine via a methyl transfer chain which is strictly dependent on B_12_. Methionine serves as an important and universal donor for methyl units via the S-adenosylmethionine (SAMet)/adenosylhomocysteine (SAHcys) cycle for various acceptors (e.g., lipids, DNA etc.). On the other side, methylene-THF serves as a one-carbon donor for the synthesis of deoxythymidine-monophosphate (dTMP) from deoxyuracil-monophosphate (dUMP) catalyzed by thymidylate synthase. Thymidine is an absolute essential building block for the synthesis of DNA. In case of severe B_12_ deficiency, methyl-THF accumulates and the folate cycle is blocked. Thus, B_12_ deficiency may lead to a secondary and functional folate deficiency (“folate trap”). DHF indicates dihydrofolate.

The first strictly B_12_-dependent enzyme is methionine synthase, which is essential for the formation of methionine. This enzyme catalyzes the transfer of the methyl unit from methyl-tetrahydrofolate (methyl-THF) to homocysteine, yielding methionine via a two-step reaction (Figure 3). In the first step, the methyl group from methyl-THF is transferred to B_12_, thus forming methyl-B_12_ and releasing THF. In the second step, the methyl group from methyl-B_12_ is transferred to homocysteine, yielding methionine. In turn, methionine transfers its methyl-unit via the S-adenosylmethionine/S-adenosylhomocysteine cycle to numerous metabolites and macromolecules for methylation of, e.g., lipids, proteins and DNA. Methylation of DNA is important for epigenetic regulation of gene expression. Methyl-B_12_ receives its methyl-unit from methyl-THF provided from methylene-THF in the folate cycle, indicating that both the cobalamin and the folate metabolism are tightly interrelated. The folate cycle provides methylene-THF for the synthesis of thymidine. Since B_12_ deficiency leads to impaired conversion of homocysteine to methionine, elevated plasma homocysteine levels may indicate functional B_12_ deficiency.

The second strictly B_12_-dependent human enzyme is methylmalonyl-CoA mutase, which is essential for the conversion of methylmalonyl-CoA to succinyl-CoA and subsequently to succinate, a common intermediate of the tricarboxylic acid cycle. This enzyme is at the end of an important biochemical degradation chain of propionyl-CoA arising during the catabolism of the amino acids methionine, isoleucine, threonine and valine, and odd chain fatty acids. After carboxylation by propionyl-CoA carboxylase, methylmalonyl-CoA is formed, which is the substrate for methylmalonyl-CoA mutase. This strictly B_12_-dependent enzyme contains one mol of adenosyl-B_12_ per subunit [12] and is located in the mitochondrial matrix. If the activity of methylmalonyl-CoA mutase is absent or decreased, e.g., due to hereditary defects of the apoprotein or to B_12_ deficiency, toxic methylmalonic acid (MMA) is formed and the excess of methylmalonic acid is excreted in the urine. The full clinical expression of methylmalonyl-CoA mutase deficiency is seen in the “inherited methylmalonic aciduria” of newborns. In any case, elevated urinary excretion or elevated serum levels of methylmalonic acid serve as specific laboratory biomarkers for functional B_12_ deficiency (see Section 4.1).

### 2.5. Clinical Pathophysiology of B_12_ Deficiency

B_12_ deficiency may manifest with hematological abnormalities and neurological symptoms. The classic hematological abnormality in B_12_ deficiency is megaloblastic anemia, possibly accompanied by leukocytopenia and/or thrombocytopenia. All of them can be fully explained from the physiologic role of B_12_ as a cofactor of methionine synthetase. In the absence of sufficient B_12_, methyl-THF accumulates (Figure 3) in the cytosol (where methionine synthetase resides) and subsequently in the nucleus. Palmer et al. [33] showed that cytosolic B_12_ depletion causes nuclear 5-methyl-THF accumulation. Methyl-THF cannot be converted back to methylene-THF because this reaction is not reversible (Figure 3). In this way, the folate cycle is blocked and a functional folate deficiency develops (“folate trap”) [3,34]. The reduced methylene-THF leads to impaired thymidine biosynthesis. Thymidine is an absolute essential building block for the synthesis of DNA. A shortage of thymidine leads to genome instability and a slow-down or even blockade in cell proliferation, which is most obvious in fast dividing cells such as bone marrow cells. This mechanism represents the background for the evolution of both megaloblastic/pernicious anemia and leukocytopenia and thrombocytopenia.

In contrast to the hematological abnormalities, the neuropsychiatric symptoms of B_12_ deficiency cannot be fully explained by the function of B_12_ as a cofactor of either of the two above-mentioned enzymes. Previous reports suggest that B_12_ deficiency-associated dysfunction of methionine synthase reduces the availability of methionine and subsequently S-adenosyl-methionine, which is an important methyl group donor (Figure 2). Transfer of methyl groups to various metabolites such as lipids, proteins (e.g., histones) and nucleic acids (e.g., DNA) has been described for more than 100 biochemical reactions [35]. The neurological symptoms would then result from reduced methylation of neuronal lipids and neuronal proteins, such as myelin basic protein, which makes up approximately one-third of the myelin of peripheral nerves and the spinal cord [36,37,38]. However, this assumption is actually far from proven [39]. Reduced genomic DNA methylation leading to defects in gene regulation was found in diabetic neuropathy (see Section 3.2). An even less likely theory suggests dysfunction of methylmalonyl-CoA mutase, which as an important detoxifying enzyme, is highly expressed in most tissues, including the nervous system [40]. Accumulation of MMA in the CNS would then be responsible for some of the neurological or psychiatric symptoms of B_12_ deficiency. In this respect, the new findings about the intracellular redox cycle of B_12_ (see Section 2.2) and its possible action as a cellular antioxidant (see Section 3.3) may provide a better explanation for the neurological symptoms of B_12_ deficiency than its role as cofactor of the two enzymes [41,42]. 

## 3. Involvement of B_12_ Deficiency in the Development of Diabetic Neuropathy

- Biochemical pathways leading to diabetic neuropathy;
- Common pathophysiological pathways of B12 deficiency-induced neuropathy and diabetic neuropathy;
- B12 may act as an intracellular antioxidant.

As mentioned before, the neurological symptoms of B_12_ deficiency are similar to those arising in the course of diabetic neuropathy. If any, the only difference is that sole sensory neuropathy occurs more frequently in B_12_ deficiency. However, B_12_ deficiency may also cause motoric or painful neuropathy, symptoms that are common in DPN. The most prevalent forms of diabetic neuropathy are peripheral, autonomic and painful neuropathy. Notably, in 10–18% of patients, nerve damage is already present at the time of diagnosis of diabetes, and neuropathy has been shown to occur even in the prediabetic state [5,43,44], which suggests that there may also be other factors contributing to neuropathy besides glucose levels or the diabetes state itself. Since B_12_ deficiency is quite common in type 2 diabetic patients (see Section 2.1) and since B_12_ deficiency causes neurological symptoms similar to DPN, B_12_ deficiency may cause the neuropathy occurring during the course of diabetes or just accelerate the progression of DPN [9]. In this case, B_12_ supplementation may help to prevent or reduce the development of DPN.

### 3.1. Biochemical Pathways Leading to Diabetic Neuropathy

Numerous molecular mechanisms leading to diabetic autonomous and sensory peripheral neuropathy have been suggested. The respective literature was recently reviewed [4,5,45]. The proposed pathomechanisms include the hyperglycemia-induced elevated sorbitol and glucosamine pathway, dysregulated glycolysis, and mitochondrial oxidative phosphorylation in diabetic nerve tissues leading to mitochondrial dysfunction and oxidative stress [46]. Numerous reports indicate an increased presence of reactive oxygen species (ROS) in DPN [47,48,49,50].

Similar to the formation of HbA1c, chronic hyperglycemia also increases the glycation of other body proteins. Long-living proteins, however, undergo further “aging”, yielding numerous protein-bound products called AGE. These elevated AGE lead to an increased binding to and stimulation of the specific receptor for AGE (RAGE), thereby activating inflammatory pathways. Earlier studies showed that deletion of RAGE improved pain perception in diabetic mice [51]. This group and others have shown that the High Mobility Globulin Box 1 Protein (HMGB1) is involved in the activation of RAGE in the RAGE pathway [1,2,52]. Recent studies illustrated roles for HMGB1 in painful DPN as treatment of diabetic mice or rats with the HMGB1 inhibitor glycyrrhizin improved mechanical and thermal pain thresholds [53,54]. Together, the suggested elevated glucose-induced mechanisms finally enhance inflammatory signal pathways and elevate oxidative stress mediated by ROS in neural cells [47].

### 3.2. B_12_ Deficiency-Induced Neuropathy and Diabetic Neuropathy Share Common Pathophysiological Pathways

While these glucocentric pathways are all based on elevated glucose levels and enhanced glucose metabolism, they do not fully explain why neuropathy may occur very early in the course of diabetes or in the prediabetic stage. There are some data suggesting that B_12_ deficiency may be a significant contributor. Straightforward evidence for a causal involvement of insufficient B_12_ supply in DPN comes from experimental animals [55]. This group has generated a mouse lacking the Cd320 receptor, which is responsible for the cellular uptake of HoloTC. These mice develop B_12_ deficiency in the nervous system and mild anemia. The structural pathology in the spinal cord presented as loss of myelin in the axonal tracts with inflammation. The sciatic nerve showed increased nonuniform, internodal segments, suggesting demyelination and remyelination in progress. Consistent with these changes, the Cd320^−/−^ mouse showed an increased latency to thermal nociception. Further, a lower amplitude of compound action potential in the sural nerve suggested that the functional capacity of the heavily myelinated axons was preferentially compromised, leading to loss of peripheral sensation. These changes resemble the clinical phenotype seen in human DPN, thus providing a strong argument that neural tissue B_12_ deficiency may be causally involved in the development of DPN. 

There are also human data indicating a significant contribution of B_12_ in DPN. First, treatment with B_12_ showed positive effects on DPN in several clinical trials [10,56,57,58,59,60,61,62,63,64,65,66,67], but not in all. For instance, one recent review found no evidence that the use of oral B_12_ supplements is associated with improvement in the clinical symptoms or in the electrophysiological markers of diabetic neuropathy [63]. However, this review comprised only 4 studies with 363 patients. Second, recent studies point to common pathology/pathophysiological mechanisms. For instance, a recent report shows a low level of genomic DNA methylation in DPN of type 2 diabetic patients but not in other complications of diabetes, such as diabetic retinopathy or nephropathy, indicating that a reduced level of genomic DNA methylation is a relatively specific risk factor for DPN [68]. Similarly, when investigating DNA methylation and gene expression in human sural nerve biopsies, Guo et al. found that DNA methylation is a mechanism in the regulation of gene expression in DPN [69,70]. Considering that reduced DNA-methylation can be one of the consequences of B12-deficiency (see Section 2.4), these data provide a mechanistic explanation of how B12-deficiency may be involved in the pathogenesis of DPN. However, to date, a precise molecular mechanism causally linking impaired methylation to DPN has not been shown. In addition, the influence of B12 treatment on the DNA methylation pattern and subsequent biological alterations has not been studied in humans yet.

Even stronger support for an important role of B_12_ deficiency in neuropathy is provided by a series of immunohistochemical studies. Haslbeck and coworkers investigated the presence of ROS and inflammatory markers in sural nerve biopsies obtained from patients with diabetic or other causes of neuropathy. They found elevated ROS biomarkers and an AGE-mediated activation of proinflammatory pathways, as indicated by an activation of the AGE-RAGE-NFκBp65 pathway in DPN [71]. In most cases, staining of proinflammatory and ROS biomarkers were colocalized in epineural vessels, perineural cells and in Schwann cells. In patients with impaired glucose tolerance and DPN, the staining patterns of sural nerves were similar but less intense compared with those found in patients with overt diabetes, indicating that ROS production and proinflammatory pathways are activated even before chronic hyperglycemia occurs [42]. To evaluate the specificity of the effects, different forms of peripheral neuropathies were studied. The authors observed a striking similarity between diabetic and B_12_ deficiency-induced neuropathy, while the staining patterns were different or absent in sural nerve biopsies from patients with other neuropathies (monoclonal gammopathy of unknown significance, Charcot–Marie–Tooth disease, idiopathic peripheral neuropathy and alcohol abuse-induced neuropathy), indicating that the pathogenesis of these neuropathies may be different [41]. Further evidence that B_12_ deficiency may lead to mitochondrial dysfunction and oxidative stress is provided by an extensive investigation by Luciani et al. [72]. They found that reduced methylmalonyl-CoA mutase activity and subsequently elevated cellular MMA levels cause extensive mitochondrial dysfunction, including abnormal mitochondrial networks, dysfunctional bioenergetics and increased oxidative stress, as shown by elevated ROS production. Treatment with two different mitochondria-targeted antioxidants normalized mitochondrial ROS production, dysfunction and structural alterations. This finding suggests that reduced B_12_ bioavailability may lead to reduced mitochondrial methylmalonyl-CoA mutase activity and increased oxidative stress and that specific treatment may rescue mitochondrial integrity. Together, the present data suggest that the development of DPN is due to mitochondrial dysfunction leading to increased local ROS production and that this derangement can possibly be prevented with suitable drug treatment. 

### 3.3. Evidence for a Role of B_12_ Acting as an Intracellular Antioxidant

While the molecular mechanisms of the essential need of B_12_ for the activity of methylmalonyl-CoA mutase and methionine synthase are broadly documented, there is little information on the molecular properties allowing B_12_ to serve as an antioxidant. As previously reviewed, the cobalt oxidation state in the center of B_12_ can vary substantially during B_12_ trafficking and processing in the cytosol and the mitochondria (Figure 2) [12]. In vitro studies showed that B_12_ exerts superoxide dismutase activity, i.e., B_12_ can react with superoxide (O_2_^−^), producing H_2_O_2_ at high rates approaching the reaction rates of the physiological defense enzyme superoxide dismutase [73]. In this reaction, superoxide oxidates Co^1+^ to Co^3+^, which can be reduced back to Co^2+^ in the cellular environment; thus, a catalytic cycle is formed. In human aortic endothelial cells, this group showed that B_12_ protects against superoxide-induced cell injury [74]. Recent in vitro and in vivo studies indicate that B_12_ may function as an endogenous antioxidant in neuronal cells [75], thus supporting a novel role for B_12_ acting as an intracellular antioxidant [76]. 

In a previous report, the current knowledge of human studies supporting the antioxidative potential of B_12_ was systematically reviewed [77]. Six studies found evidence for antioxidative properties of B_12_, the results of four studies remained unclear, and the results of one study did not support any antioxidative properties of B_12_. The potential antioxidant properties may act at several stages: (i) by directly scavenging ROS, (ii) indirectly by preserving high glutathione levels, (iii) by reducing oxidative stress induced by metabolic pathways, and (iv) by modulation of cytokine production. Possibly, the suggested pathways may act, at least in part, in common. In a diabetic rat model for DPN, Mizukami et al. found that treatment with methyl-B_12_ prevented activation of protein kinase C in nerve tissue and reduced the diabetes-induced increased number of 8-oxo guanosine positive cells in the endoneurium [78]. Since 8-oxo guanosine is an integrative marker for oxidative stress, these results indicate that administered pharmacological B_12_ preparations may help to prevent the diabetes-induced increase in ROS in peripheral nerves.

Taken together, these findings suggest a causal role for B_12_ deficiency in different forms of neuropathies including DPN, very likely through mitochondrial dysfunction and oxidative stress/ROS formation, and support the possibility that B_12_ acts as a cellular antioxidant in subclinical forms of functional B_12_ deficiency, independent of the classical mechanisms of B_12_ serving as a cofactor. This notion is supported by the fact that B_12_ supplementation may improve clinical symptoms regardless of the actual serum B_12_ status [79]. Wolffenbuttel et al. suggest that “B_12_ may not be only a vitamin in the general sense, but rather a general nerve protecting and nerve regenerating factor particularly in DPN” [79]. However, although numerous aspects indicate the beneficial action of B_12_ for the prevention or treatment of (sub-)clinical DPN, there are no reports in the literature unequivocally linking B_12_ treatment with its beneficial effects on DPN on a molecular basis. Therefore, concrete evidence, particularly in humans, is still lacking.

## 4. Laboratory Determination of B_12_ Deficiency

- Biomarkers of B_12_ deficiency;
- Laboratory biomarkers and decision limits for B_12_ deficiency in the general population;
- B_12_ deficiency in the elderly population: special considerations and decision limits.

### 4.1. Biomarkers of B_12_ Deficiency

Because, as mentioned above, clinical symptoms and signs may be subtle or absent in cases of subclinical B_12_ deficiency, the diagnosis has to be supported by determination of laboratory biomarkers [14,79]. The following parameters are available for assessing B_12_ adequacy or deficiency: serum (total) B_12_, HoloTC, homocysteine (HCys) (Figure 3), and methylmalonic acid (MMA) (Figure 2). 

Serum B_12_ and HoloTC concentrations are direct biomarkers for the estimation of the body’s B_12_ supply. Among them, the determination of B_12_ serum concentration is most widely used in routine clinical practice to document the presence of B_12_ deficiency. However, concentrations of total serum B_12_ do not adequately mirror the actual supply of B_12_ in the human body. Rather HoloTC, which reflects the biological form of the body’s B_12_ supply, is the most sensitive biomarker for early B_12_ deficiency [80]. One study using receiver operator curves (ROC) for the comparison of the two biomarkers determined in 2403 individuals found that that HoloTC was superior compared with conventional B_12_ measurements for diagnosing B_12_ deficiency [81]. HoloTC had a greater area under the ROC compared with serum B_12_ concentrations (0.85 vs. 0.76) in all participants. Similarly, Gwathmay and Grogan [82] report that HoloTC is a more sensitive biomarker for B_12_ deficiency. In another study, Herrmann et al. [83] found values of 0.879 vs. 0.836 for HoloTC and total B_12_, respectively, supporting the notion that HoloTC is modestly superior compared with serum B_12_ concentrations. However, for the sake of completeness, we also note that there is also a single reference that HoloTC is not superior to total B_12_ [84].

An increase in the metabolites homocysteine (HCys) (Figure 3) and methylmalonic acid (MMA) (Figure 2) is considered to mirror cellular deficiency of B_12_, also termed “functional B_12_ deficiency”. Nevertheless, they seem not to be valuable as screening tests. For instance, in the aforementioned study of Herrmann et al. [83], which included 111 vegetarian subjects, HoloTC was found to be the earliest biomarker for B_12_ deficiency followed by MMA, and serum B_12_ was the latest biomarker. Most importantly, however, mainly MMA and, to a lesser degree, HCys concentrations were influenced by renal function. In a cohort of 1143 apparently healthy elderly Swiss participants >60 years, HCys and MMA levels were influenced by renal function as estimated by eGFR [85]. In this extensive study, the authors found an age-dependent increase in HCys and MMA concentrations, which were inversely related to eGFR, indicating that not age per se, but rather the age-dependent decrease in GFR is responsible for the increase in MMA and HCys when GFR is below 36 mL/min. Notably, both B12 and HoloTC were unrelated to eGFR. Taken together, the present data suggest that HoloTC is a more sensitive biomarker for the detection of insufficient B_12_ supply than any other biomarker and that modestly decreased HoloTC may also indicate the presence of early cellular or functional B_12_ deficiency. MMA and HCys levels can also be useful for differentiation of B_12_ from folate deficiency: normal MMA and increased homocysteine levels are consistent with folate deficiency, and conversely, increased MMA levels without elevated folate levels indicate B_12_ deficiency.

### 4.2. Laboratory Biomarkers for B_12_ Deficiency in the General Population and Decision Limits

From the previous discussion on the available biomarkers (Section 4.1) it becomes apparent that no single biomarker is capable of covering all possible aspects of B_12_ deficiency (screening, subclinical/cellular deficiency, clinically overt deficiency). Furthermore, there is no consensus in the literature with regard to the cut-offs for each of the above-mentioned biomarkers. Reviewing 69 publications on cut-off points for the diagnosis of B_12_ deficiency in the general population [86], the authors report on broad ranges of decision limits: 100–350 pmol/L for serum total B_12_; 20–50 pmol/L for HoloTC; 0.21–0.47 µmol/L for methylmalonic acid; and 10–21.6 µmol/L for homocysteine. These authors conclude that it is necessary to establish different reference cut-offs according to age and the analytical method used. It is, therefore, not surprising that for the screening of B_12_ deficiency, numerous algorithms have been developed [1,87].

**Figure 4:**
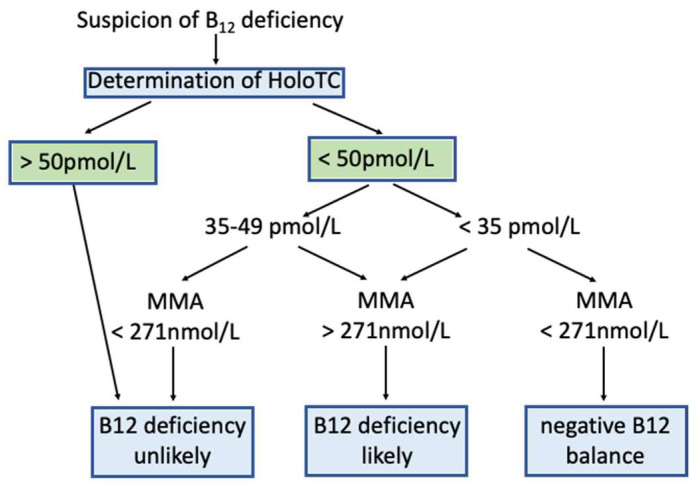
Algorithm for the detection of B12 deficiency. An algorithm for the detection of B_12_ deficiency is shown (adapted and modified from Herrmann and Obeid [88]). This algorithm suggests that HoloTC be used for screening of B_12_ deficiency. For further differentiation, determination of serum MMA is used, provided that there is no severe renal insufficiency. For details see text (Section 4.1 and Section 4.2).

We suggest here an algorithm for practical application based on a suggestion of Herrmann et al. (modified from [88], Figure 4). Inevitably, the diagnosis of B_12_ deficiency is probabilistic, i.e., “(very) likely”, “possible” or “unlikely”. Because HoloTC is the most specific and most sensitive laboratory biomarker for the detection of B_12_ deficiency, HoloTC should be used for screening. If serum levels of HoloTC are above 50 pmol/L, B_12_ deficiency is very unlikely. If serum levels of HoloTC are below 35 pmol/L and MMA levels are above 271 nmol/L, B_12_ deficiency is likely. If serum levels of HoloTC are below 35 pmol/L and MMA levels are below 271 nmol/L, a negative B_12_ balance is likely, i.e., there is no cellular B_12_ deficiency but an insufficient B_12_ supply. If serum levels of HoloTC fall into the range of 35–49 pmol/L, MMA values above 271 nmol/L suggest B_12_ deficiency, and if they are below 271 nmol/L B_12_, deficiency is unlikely. In any case renal dysfunction should be excluded. 

### 4.3. B_12_ Deficiency in the Elderly Population

The situation in elderly people deserves particular consideration [1,2,14,87,89,90]. From a clinical point of view, some disorders occur in the elderly more frequently than in younger patients. These include the so-called “Food cobalamin malabsorption” (see Section 2.1) [91] and the classic autoimmune atrophic gastritis, as well as diabetes and cardiovascular disease, the latter often being treated with medications potentially leading to B_12_ malabsorption (Section 2.1). In these cases, B_12_ deficiency is mostly only moderate. Furthermore, clinical symptoms are usually mild or even absent in the elderly. It follows that B_12_ deficiency is in many cases subclinical or “functional”, and there is an imperative need for laboratory biomarkers to set or support the diagnosis as early as possible. However, laboratory diagnosis faces two issues. First, in most studies, age appears to be an influence factor for laboratory biomarkers of B_12_ deficiency [81,83,86]. However, as mentioned before, several other studies show that not age per se but rather age-related renal insufficiency may increase serum levels of HCys and particularly MMA since GFR declines with age [83]. Thus, the usefulness of increased HCys and MMA, generally considered to be markers of “functional” B_12_ deficiency, is limited in the elderly. In a comprehensive study (n = 1143), HoloTC performed better (AUC = 0.923) than B_12_ (AUC = 0.884) and appeared to be the superior biomarker for screening of B_12_ deficiency, also in elderly people above 60 years (and up to slightly more than 80 years) [85]. Second, since specific symptoms of B_12_ deficiency are usually very mild or absent, the question arises whether decision limits for treating (suspected) B_12_ deficiency should be set higher in the elderly than in younger patients in order to enhance the sensitivity of each biomarker. Based on their results, the authors of the aforementioned study [85] provided a suggestion for decision limits, as shown in Table 2. It is important to note that the B_12_ values suggesting sufficient B_12_ supply are much higher than those suggested by other authors and also higher than the values suggested by the WHO (which are shown in the table for comparison). For instance, the respective cut-offs for total serum B_12_ levels are 316 vs. 221 pmol/L (Table 2). These data suggest that B_12_ decision levels need to be higher in elderly people to ensure sufficient B_12_ supply and to avoid cellular B_12_ deficiency.

### 4.4. Summary of Laboratory Assessment of B_12_ Deficiency

Although a gold standard for diagnosing B_12_ deficiency is lacking [92], laboratory biomarkers are valuable for screening, for the diagnosis of B_12_ deficiency and for therapeutic monitoring of B_12_ deficiency. While B_12_ levels are widely used, HoloTC levels appear to react earlier and appear more sensitive for documenting B_12_ deficiency. Although the biomarkers MMA and HCys may indicate functional B_12_ deficiency in tissues, they are not valuable as screening tests since they are less sensitive and are influenced by renal function (Table 2). MMA can also be useful for differentiation of B_12_ from folate deficiency. Subclinical and overt B_12_ deficiency may be easily overlooked in the elderly population since clinical signs are absent or nonspecific, laboratory biomarkers may be influenced by reduced kidney function, and B_12_ absorption is impaired by reduced gastric acidity, e.g., due to proton-pump inhibitors or other drugs, e.g., metformin.

## 5. Conclusions

B_12_ is an essential cofactor for two important enzymes in mammals. Besides its function serving as a cofactor, recent findings suggest that the central Co atom of B_12_ undergoes several reduction/oxidation steps ranging from 1+ to 3+, both in the cytosol and in mitochondria. The recognition that B_12_ exerts intrinsic antioxidative activity indicates a new role of B_12_ in cellular metabolism beyond its classical, well-known cofactor function. Since several studies point to an involvement of oxidative stress in the development of DPN, the antioxidative potential of B_12_ suggests that B_12_ administration may be justified, not only for the therapy of overt DPN, but also in early stages of DPN, or even for prevention of DPN when cellular oxidative stress is present but symptoms are mostly absent. The absence of a clinical improvement after B_12_ administration in some studies does not exclude a possible favorable effect of B_12_ because oxidative stress is probably only one of the multiple mechanisms leading to DPN. Of course, further studies are needed to elucidate in more detail the complex physiological action of B_12_ and to prove the putative favorable action of B_12_ administration, particularly in the early stages of DPN.

## Figures and Tables

**Table 1:** Stages of B_12_ absorption and metabolism and possible defects leading to B_12_ deficiency.

Stages of B_12_ Metabolism	Defects/Causes of B12 Deficiency
**1. Dietary uptake:** B_12_ present in free or protein-bound form is taken up in the diet or as a drug.	Inadequate intake (strict vegan diet, eating disorders etc.).
**2. Gastric secretion of intrinsic factor (IF) and HCl:** In the stomach, the protein-bound B_12_ complex is digested by pepsin in the acid milieu, and the free B12 is bound by haptocorrin. This complex and IF (secreted by parietal cells) are transferred to the duodenum.	*Impaired secretion or neutralization of HCl* • Elderly people • Drugs, e.g., proton pump inhibitors, histamine receptor antagonists *Impaired secretion of HCl and IF* • Autoimmune gastritis/gastric atrophy (antibodies against parietal cells or IF) • Gastrectomy • Hereditary defects of IF
**3. Binding of B_12_ by IF in the duodenum:** In the duodenum, the B_12_-haptocorrin complex is digested by proteases, and the released B_12_ is bound by IF with high affinity.	
**4. Absorption in ileum:** The B_12_–IF complex is bound to and taken up by specific mucosal receptors in the terminal ileum.	Malabsorption due to, e.g., pancreas insufficiency, surgery, inflammatory bowel disease, or drugs, e.g., biguanides (metformin)
**5. Blood-borne transport by transcobalamins:** After internalization by the enterocytes, B_12_ is exported into the blood and subsequently bound to transcobalamins (TC). The complex with holotranscobalamin (HoloTC) carries 10–20% of total B_12_. HoloTC is the circulating form of B_12_ which can be taken up by the target cell via the ubiquitous receptor CD 320. The majority of B_12_ is transported by TC I, but this B_12_ complex cannot be taken up by peripheral cells.	Congenital defects (very rare in adults)
**6. Intracellular transport and lysosomal metabolism:** The internalized complex is transported to the lysosomes and degraded, thereby liberating B_12_. Free intracellular B_12_ can be metabolized into methylcobalamin or adenosylcobalamin, both being cofactors, the first for methionine synthase and the second for methylmalonyl-CoA mutase.	Congenital defects (very rare in adults)

**Table 2:** Laboratory biomarkers for estimation of B_12_ deficiency in the elderly population [85] and, for comparison, the corresponding B_12_ values suggested by the WHO [87].

Biomarker	B12 Deficiency	Low/Grey Zone	Adequate Supply
**B_12_ ^1^ (pmol/L)**	<131	131–315	≥316
**HoloTC (pmol/L)**	<25.8	25.8–56.9	≥57
**MMA ^2^ (µmol/L)**	>0.485	0.217–0.485	≤0.216
**HCys ^2,3^ (µmol/L)**	>26.8	9.6–26.8	≤9.5
***WHO***			
**B_12_ ^1^ (pmol/L)**	<148	148–220	≥221

**HoloTC**: holotranscobalamin; **MMA:** methylmalonic acid; **HCys**: homocysteine. ^1^ May be determined in serum or plasma [80]. ^2^ Serum or plasma. Nota bene: HCys and MMA are elevated when renal function declines. For screening of inborn errors of metabolism, MMA is determined in urine. ^3^ HCys determination only in plasma.

## Data Availability

Not applicable.
